# Combination treatment with whole body vibration and simvastatin improves the early osseointegration in aged rats

**DOI:** 10.1016/j.bonr.2024.101790

**Published:** 2024-07-15

**Authors:** Zheng-Bo Qiao, Ming-Zhong Gu, Yu-Wu Wang, Bin-Bin Ma, Shan-Shan Pang

**Affiliations:** a Department of Emergency Medicine, The Yancheng School of Clinical Medicine of Nanjing Medical University, Yancheng Third People's Hospital, Yancheng 224000, China; b Department of Orthopedics, The Yancheng School of Clinical Medicine of Nanjing Medical University, Yancheng Third People's Hospital, Yancheng 224000, China; c Department of General Medicine, The Yancheng School of Clinical Medicine of Nanjing Medical University, Yancheng Third People's Hospital, Yancheng 224000, China

**Keywords:** Bone loss, Osseointegration, Oxidative stress, Whole body vibration, Simvastatin

## Abstract

**Background:**

Current research has demonstrated that Simvastatin (SIM) and Whole Body Vibration (WBV) actively contributes to the repair of osteoporotic bones. However, there is still limited knowledge regarding the impact of this combined therapy on osseointegration in elderly individuals. Objective: The objective of this study was to verify the influence of WBV and SIM combination treatment on Titanium implants' fixation strength in aged rats.

**Methods:**

Male Sprague-Dawley rats at 24 months old were utilized for this investigation. Titanium rods were surgically inserted into the distal femoral canal on their left side. Subsequently, all animals were randomly assigned to one of four groups: Control group; WBV group; SIM group; and WBV + SIM group. Each group received Saline, Whole Body Vibration, Simvastatin, or a combination of Whole Body Vibration plus Simvastatin treatment until they reached their natural death after 12 weeks. The bilateral femurs and serum samples from these rats were collected for evaluation purposes.

**Results:**

Both WBV and SIM treatments exhibited an increase in bone mass, osseointegration, and push-out force compared to the Control group (all, *P* < 0.05). Additionally, levels of oxidative stress and inflammatory factors decreased with both treatments when compared to the Control group alone (all, *P* < 0.05). Notably, the WBV + SIM group displayed superior effects on new bone formation, biomechanical strength, BMP2 expression in bone tissue as well as SOD2 expression regulation related to bone repair genes when compared to other groups involved in this study (all, *P* < 0.05).

**Conclusion:**

These findings suggest that combining physiotherapy (WBV) with drug therapy (SIM) proves beneficial for enhancing implant fixation in aged rats.

## 1 Introduction

Revision total joint replacement may become necessary due to various factors affecting implant fixation despite the overall success and widespread use of this orthopaedic procedure (Zhou and Tao, 2022). Implant support can be compromised by reduced bone mass caused by conditions such as osteoporosis - a chronic ailment characterized by weakened bones resulting from diminished bone density and structural integrity within the skeletal system (Marini et al., 2023; Sindel, 2023). Addressing this issue requires implementation of effective strategies. An alternative method involves enhancing implant surface characteristics through utilization of coatings composed of biologically active substances (Tao et al., 2016a; Tao et al., 2016c). Increasing the effectiveness of multiple drug combinations is a feasible option (Chen et al., 2018; Tao et al., 2015), but this inevitably leads to an increase in side effects. However, although the modification of the material itself improves the osteogenic activity of the joint prosthesis, the loss of bone mass due to increasing age does not improve, thus prompting us to explore new solutions to solve this situation.

To enhance the successful integration of implants in elderly patients, it is beneficial to select an effective intervention. Statins are commonly used inhibitors of 3-hydroxy-3-methylglutaryl-coenzyme reductase, traditionally employed for reducing cholesterol production in cardiovascular diseases (Kouhpeikar et al., 2022). Moreover, research has indicated their potential in promoting bone regeneration through a dual mechanism: improving osteogenesis and inhibiting osteoclast activity (Lin et al., 2022). Studies have suggested that simvastatin (SIM) can increase bone mineral density (BMD), reduce the occurrence of osteoporotic fractures, and facilitate fracture healing by positively influencing bone metabolism and enhancing osseointegration in models with bone loss (Moshiri et al., 2016; Zhou-Shan et al., 2020). Another non-pharmacological intervention called low-magnitude high-frequency mechanical stimulation, such as whole body vibration (WBV), has demonstrated significant anabolic properties by increasing both BMD and bone strength while decreasing bone turnover rates in ovariectomized rats and postmenopausal women (Tsartsalis et al., 2012). Interestingly, recent experimental findings have supported WBV as a helpful approach for improving osseointegration under conditions of osteoporosis by regulating the lifespan of osteoblasts positively and suppressing the activity of cells resembling osteoclasts (Shobara et al., 2021; Zhou et al., 2015).

While combination therapy is commonly used in clinical practice, the evident benefits of combining SIM with parathyroid hormone (1–34) are apparent(Tao et al., 2016b). Considering the expensive cost and potential side effects associated with current antiosteoporosis drugs, WBV treatment may offer a more promising alternative approach to enhance osseointegration due to its affordability, safety, and noninvasive nature. This gives us a possible hypothesis: the feasibility of a drug combined with physical therapy to promote osseointegration in osteoporosis. However, there is a lack of research on the combined effects of SIM and WBV in improving osseointegration. Therefore, in this study, we used a titanium rod implantation model in aged rats, and observed that WBV combined with SIM intervention had better effects than intervention alone, as assessed by imaging, biomechanical, and histological evaluation.

## 2 Materials and methods

### 2.1 Experimental animals

Sixty male Sprague Dawley rats, aged 24 months and weighing approximately 530 ± 26 g, were enrolled for the purpose of this study. In groups of four, the animals were gradually accustomed to a standard cage environment with a balanced light-dark cycle lasting 12 h each, while being provided unrestricted access to water and rodent diet. During the course of this investigation, adherence to the Committee for the Update of the Guidelines for the Care and Use of Laboratory Animals (Bielitzki et al., 2011) was carefully observed. Furthermore, ethical clearance was duly secured from the Yancheng Third People's Hospital Animal Experiments Local Ethics Committee (Approval No: 2024-2-32) in support of this research study.

### 2.2 Study design

The titanium implants (manufactured by Guangzhou Huachuang medical equipment Co., LTD) were bilaterally inserted in all animals, featuring a 1.0 mm outer diameter and measuring 20 mm in length. A concise and suitable model for the implantation into the femoral cavity (Sun et al., 2020; Virdi et al., 2012) was utilized for this study. On the day of surgical intervention, the animals were anesthetized with a combination of Ketamine (50 mg/kg, i.p.) and Xylazine (5 mg/kg, i.p.). The medullary canal was reached by making an incision in the skin and creating an opening in the femoral condyle, after which the contents were disturbed and flushed out using a solution of saline. Following this, the implant was inserted into the medullary canal and secured by applying bone wax(Braun Medical Spain AG) to seal the aperture. Following the surgical procedure, an administration of diclofenac sodium (100 μl, i.p.) was initiated, while a course of cefpodoxime antibiotic (500 mg/kg, i.p.) was prescribed for a duration of three days.

One week after left implantation, the aged rats were divided into four groups (each group consisting of ten rats): Con, WBV, SIM, and WBV + SIM. Rats from SIM and WBV + SIM groups received simvastatin (25 mg/kg/day) via gavage administration. Rats from WBV and WBV + SIM groups were housed in cages that could be securely placed on a GJX-5 vibration sensor (Beijing Sending Technology, Beijing, China). These rats underwent vertical whole-body vibration (WBV) at a magnitude of 0.3 g and frequency of 40 Hz for 30 min every 12 h over five days per week as previously described(Zhou et al., 2011). The dosage of simvastatin was determined based on previous experiments demonstrating its protective effects in OVX rodent models(Shahrezaee et al., 2018). After twelve weeks following implantation, all rats were euthanized by cervical dislocation and serum samples along with femurs were collected from sacrificed rats for subsequent testing purposes.

### 2.3 Micro-CT evaluation

The distal femur with implants (five specimens per group) underwent scanning on a μCT system (micro-CT 40; Scanco, Wayne, Pennsylvania) and was reconstructed with an isotropic voxel size of 10 μm. The scanning system parameters were set to 70 kV, 114 μA, and 700 ms integration time in order to achieve maximal signal-to-noise ratio and optimal X-ray transmission through the titanium implant. Following the scanning process, a constrained three-dimensional (3-D) Gaussian filter (sigma = 1.2, support = 2) was applied to partially suppress the noise in the volumes. Additionally, scattering artifacts present in micro-CT images resulting from the titanium implant were remedied using a multilevel thresholding procedure. Specifically, a threshold in per mille of maximal gray value ranging from 225 to 550 represented trabecular bone; values exceeding 550 indicated cortical bone and titanium implant; while values falling below 225 were recognized as unmineralized tissue. The acquisition of three-dimensional (3-D) images served for both qualitative and quantitative evaluation purposes whereby the volume of interest (VOI), encompassing the entire trabecular compartment around each implant from slice positioned at least 2 mm below the growth plate down to distal 100 slices was considered. The trabecular bone parameters assessed in this study included the relative volume of bone compared to the total volume (BV/TV), the number, thickness, and density of trabeculae (Tb. N, Tb. Th, Conn.D), as well as measures of bone mineral density and content (BMD, BMC) within a specific area of interest(Gabet et al., 2008; Li et al., 2013).

### 2.4 Mechanical test

Following the micro-CT scan, a push-out test was conducted on the specimens (*n* = 10 per group) utilizing a universal material testing system (Instron 4302; Instron, Norwood, MA, USA). The epiphyseal separation was performed to expose the implant end. A custom-designed mould made of self-curing plastic was created for each individual specimen to maintain compression force along the long axis of the implant. During the test, a compression speed of 1 mm/min was applied and displacement versus force data were recorded for analysis of maximal push-out force and ultimate shear strength measurement(Li et al., 2010).

### 2.5 Histology

The left femora, together with the implants, were immersed in a 4 % paraformaldehyde solution and allowed to fix for a period of 24 h. Subsequently, they underwent dehydration using progressively increasing concentrations of alcohol (70 %, 95 %, and finally 100 %), with each concentration being maintained for two days. After undergoing this procedure, the samples were immersed in methyl methacrylate without undergoing decalcification. To examine the tissue characteristics within the medullary canal and at the bone-implant interface, transverse sections measuring 70 μm were obtained from the proximal half of each implant site located at the metaphysis of the femora. Utilizing a SP1600 microtome manufactured by Leica Microsystems (Wetzlar, Germany), the following sectioning procedures were performed: three sections were obtained from each femur and stained with Von-Gieson. The Bone area ratio (BAR) and Bone to implant contact (BIC) were measured on sections located approximately 2 mm below the epiphyseal plate within the entire trabecular compartment around the implant using Leica DMI 6000B microsystems (Germany), in accordance with established protocols(Li et al., 2010).

The femora without implants were decalcified using 10 % EDTA, followed by HE and Masson staining as well as immunohistochemical assay according to the provided instructions. Paraffin-embedded bone tissues were sectioned at a thickness of 5 μm. Deparaffinization was performed using an environmentally friendly de-paraffin liquid (G1128, Servicebio, China), and dehydration was carried out with gradient alcohol. The membrane-breaking solution (Servicebio, China) was utilized following the recommended protocols.

On the basis of perfect bone decalcification and sectioning, immunofluorescence staining was performed to evaluate the expression of specific proteins in bone tissue. To block endogenous peroxidase activity, the sections were incubated with 3 % BSA for 25 min before being exposed overnight at 4 °C to primary antibodies against SOD2 (abcam, diluted at 1:200) and BMP-2 (abcam, diluted at 1:100). On the subsequent day, HRP-conjugated Goat Anti-Rabbit IgG H&L (abcam, diluted at 1:300) was applied to the sections for a duration of one hour. Stained sections were visualized under light microscopy (BX43, Olympus, Japan) to capture images. The fluorescence intensity of the target protein was then selected in the 2 mm area of the growth plate and the distal end of the growth plate, and in the area between the cortical bones on both sides of the 0.42 mm^2^ area. If the area measured is different between different slices, we will normalize the fluorescence intensity per mm^2^. Finally, we select each group and each slice to generate a data for evaluation.

### 2.6 Evaluation of serum indexes

The levels of inflammatory cytokines such as interleukin (IL)-6, IL-1β, and tumor necrosis factor (TNF)-α were measured in plasma using commercially available, rodent-specific ELISA kits (Wuhan Saipei Biotechnology Co., LTD; IL-6(SP12279), IL-1β(S SP10180), TNF-α(SP12250)).The bone formation markers total alkaline phosphatase (ALP), osteocalcin (BGP), cross-linked carboxyl terminal region of type 1 collagen (CTX-1) and tartrateresistant acid phosphatase 5b (TRACP-5b) were measured in plasma using commercially available, rodent-specific ELISA kits (Wuhan Gilead Biotechnology Co., LTD; kit #s: BGP (J22898), ALP (J22897); Wuhan Saipei Biotechnology Co., LTD; CTX-1 (SP12422), TRACP-5b (SP12917)).Additionally, the levels of SOD, glutathione peroxidase (GSH-PX), and malondialdehyde (MDA) present in serum were assessed in plasma using commercially available, rodent-specific ELISA kits (Nanjing Jiancheng Technology Co., LTD; kit #s: SOD (A001-1-2), GSH-PX (A005-1-2), MDA (A003-1-2)), as the merchant's instructions described.

### 2.7 Real-time quantitative RT-PCR (RT-qPCR)

After 12 weeks of different treatments, bone tissue samples from VOI in rats were collected. Total RNA was extracted using TRIzol reagent (Invitrogen) following an established protocol(Fabien et al., 2013). The tissue samples were homogenized with liquid nitrogen using a mortar and pestle. Then, TRIzol reagent (5 ml per 50–100 mg of bone tissue) was added. After homogenization, centrifugation at 10,000 rpm for 10 min at 4 °C removed insoluble material. Next, one microgram of the obtained total RNA underwent reverse transcription into cDNA using the Prime Script TM RT reagent kit (TAKARA, Dalian, China) as per the manufacturer's instructions. To measure gene expression levels, a quantitative RT-PCR assay was performed on an ABI 7500 Sequence Detection System (Applied Biosystems, USA) with specific oligonucleotide primer sequences (summarized in Table 1). Finally, the gene expressions were normalized to GAPDH, a housekeeping gene.

**Table 1:** The nucleotide sequences for the real-time RT-PCR primers used as following.

Genes	Forward (5′-3′)	Reverse (5′-3′)
RANKL	AGCCGAGACTACGGCAAGTA	AGACCACCTGACCCAGTCC
OPG	GCTGGCACACGAGTGATG	GCAGAATTCGAGCTCCAGGTA
Runx2	TCCCAGTATGAGAGTAGGTGTCC	GGCTCAGATAAGAGGGGTAAGAC
GAPDH	ACATTGTTGCCATCAACGAC	TACTCAGCACCAGCATCACC

RANKL, receptor activator of nuclear factor-κB ligand; OPG, osteoprotegerin.

### 2.8 Statistical analysis

The relevant findings are presented as the mean value ± standard deviation (SD). Analytical approaches employed encompassed Student's *t*-test and one-way analysis of variance (ANOVA). Significance was established at *P* values below 0.05. Statistical analysis was conducted using IBM SPSS Statistics 19.0 software. *P < 0.05 vs Con, #*P* < 0.05 vs WBV, &P < 0.05 vs SIM.

## 3 Results

### 3.1 Evaluation osseointegration by Micro-CT and VG staining

**Fig. 1:**
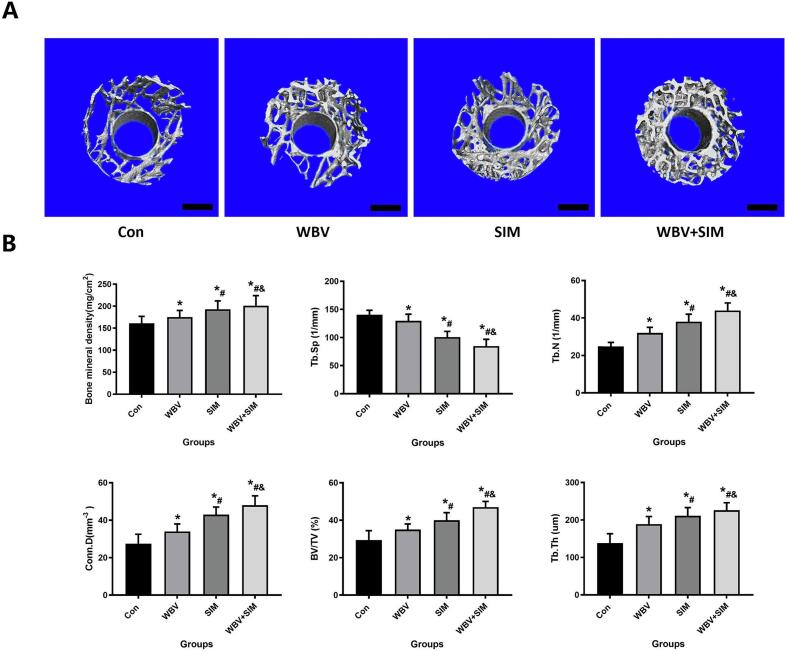
WBV plus SIM can significantly improve osseointegration in aged rats. A. Micro-CT scans were conducted on the distal femur at a 12-week interval following the placement of implants in four groups: Con, WBV, SIM, and WBV + SIM. B. Assessment of implant osseointegration at the 12-week milestone after implantation through micro-CT analysis, employing quantitative measures.

The micro-CT images clearly depicted the variations in trabecular microstructure of implant bone tissue among different groups (Fig. 1 A). The summarized results from quantitative analysis of micro-CT data can be seen in Fig. 1 B. Among all the micro-CT parameters, the combined therapy involving WBV and SIM exhibited the most significant effects. In comparison to the Con, WBV, and SIM groups, higher values for BMD, BV/TV, Tb. N, Conn.D, Tb. Th were observed in the WBV + SIM group along with a lower value for Tb.Sp. Notably, it was found that the strongest impact on BMD, BV/TV, Tb. N, Conn.D, Tb.Th, and Tb. Sp was detected in the WBV + SIM group (*P* < 0.05).

**Fig. 2:**
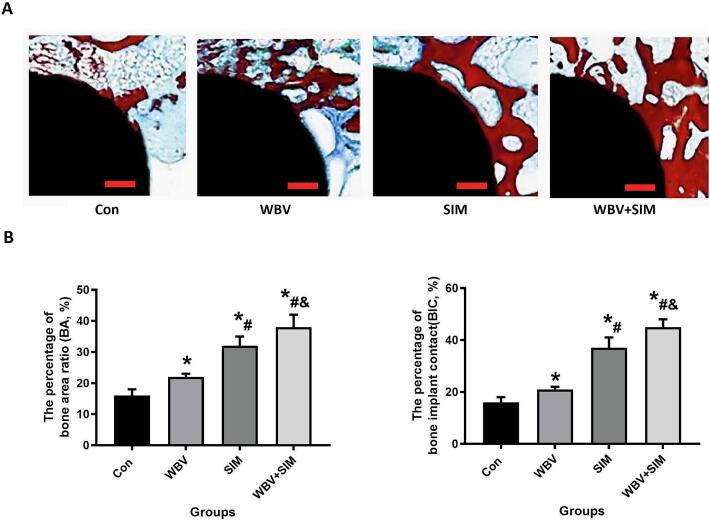
Combined treatment with WBV and SIM can significantly improve osseointegration in aged rats. A. Images of representative Von-Gieson stained sections of the distal femur 12 weeks after implantation in Con, WBV, SIM and WBV + SIM groups. B. Changes of BIC and BAR values in Con, WBV, SIM and WBV + SIM groups.

The positive impact of various interventions on implant osseointegration was observed through histomorphometric analysis of undecalcified sections with implants (Fig. 2 A). All treatments exhibited a noteworthy improvement in Bone area ratio (BAR) and Bone to implant contact (BIC) when compared to the control group (P < 0.05, as shown in Fig. 2B). At 12 weeks, the BAR calculation results for WBV, SIM, and WBV + SIM groups demonstrated a noticeable improvement compared to the control group (P < 0.05). Similarly, there was an increase in BIC contact results at 12 weeks for WBV, SIM, and WBV + SIM groups when compared to the control group (*P* < 0.05).

### 3.2 Biomechanical test

**Fig. 3:**
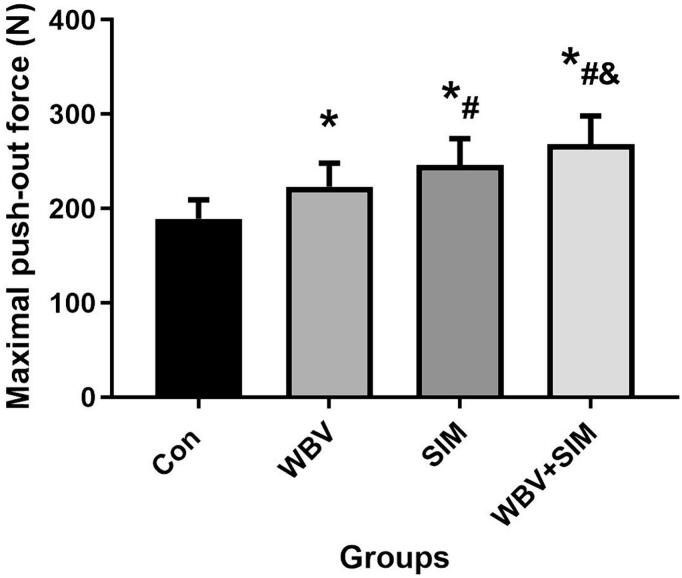
The biomechanical outcomes were quantified as the maximum force (N) required for push-out in four groups: Control group, WBV group, SIM group, and WBV + SIM group.

The WBV, SIM, and WBV + SIM groups demonstrated a noticeable improvement in the maximum push-out force compared to the Con group (*P* < 0.05). Furthermore, the maximum push-out force of the WBV + SIM group was significantly superior to that of the Con, WBV, and SIM groups. These findings suggest that administering SIM systemically may have an additional beneficial effect on enhancing implant fixation in elderly rats treated with WBV (Fig. 3).

### 3.3 Evaluation bone mass by Micro-CT, HE and Masson staining

**Fig. 4:**
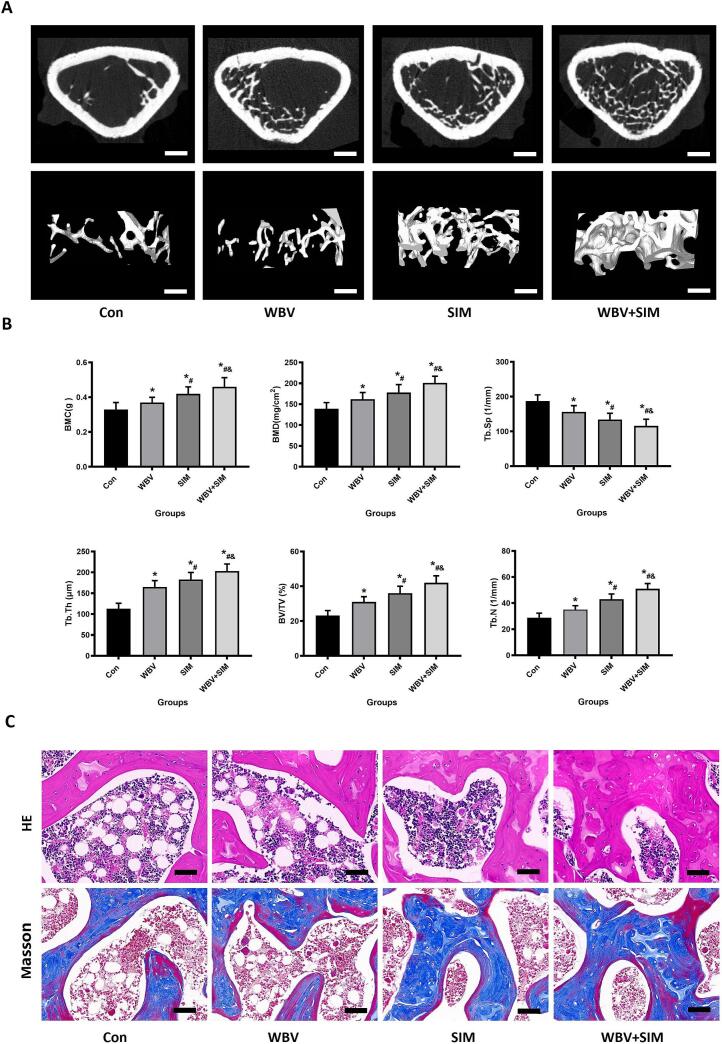
The results obtained from micro-CT and Histological evaluation were employed to demonstrate that the combination of WBV with SIM resulted in a significant increase in bone mass in OVX rats. A: Representative Micro-CT 2D and 3D reconstructions, as well as scan images after 12 weeks of treatment from the Con, WBV, SIM, and WBV + SIM groups (scale bar = 1 mm). B: The quantitative parameters including BMD, BV/TV, Tb. Th, Tb. N, Conn.D and Tb.Sp (*N* = 5). C: Systemic administration of WBV and SIM led to increased bone mass in OVX rats when evaluated using HE and Masson stainings (scale bar = 100 μm).

We further assessed the enhancement of trabeculae in the epiphysis of the femur through Micro-CT scans, HE and Masson sections, as well as staining techniques (Fig. 4). Micro-CT analysis revealed that both WBV and SIM could promote bone mass in the distal femur of aged rats, leading to significant increases in BV/TV, Tb.N, BMD, BMC, Tb.Th values and a decrease in Tb.Sp value (P < 0.05), compared to the Control group. HE and Masson staining demonstrated that both WBV and SIM were capable of improving trabecular bone quality while reducing adipocyte formation. However, we observed that combining WBV with SIM yielded superior results compared to using WBV or SIM alone.

### 3.4 Analysis of bone metabolism, oxidative stress and inflammatory factors

**Fig. 5:**
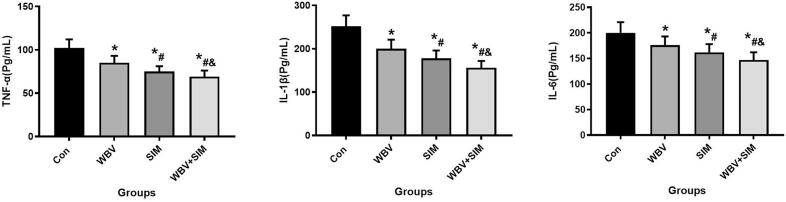
Changes in serum IL-1β, IL-6, and TNF-α at 12 weeks of intervention were observed in Con group, WBV group, SIM group, and WBV + SIM group.

**Fig. 6:**
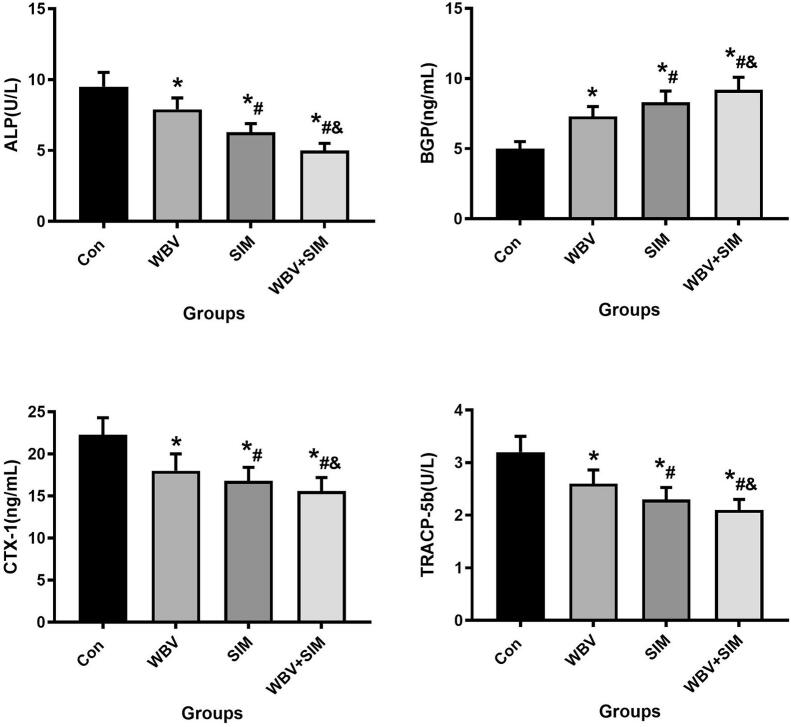
Changes in serum ALP, CTX-1, TRACP-5b and BGP at 12 weeks of intervention were observed in Con group, WBV group, SIM group, and WBV + SIM group.

**Fig. 7:**
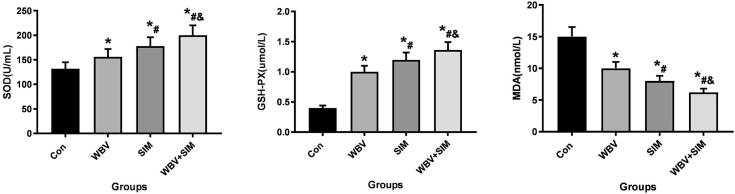
Changes in serum MDA, SOD, and GSH-PX at 12 weeks of intervention were observed in Con group, WBV group, SIM group, and WBV + SIM group.

The results depicted in Fig. 5, Fig. 6, Fig. 7 demonstrate that WBV and SIM interventions significantly reduced the levels of IL-1β, IL-6, TNF-α, ALP, CTX-1, TRACP-5b, and MDA (*P* < 0.05), while concurrently increasing the levels of BGP, SOD, and GSH-PX when compared to the Con group. Notably, the combined therapy of WBV with SIM exhibited the most favorable impact on these aforementioned indices. These findings suggest that WBV combined with SIM not only influences bone turnover but also mitigates inflammation and oxidative stress levels.

### 3.5 Immunofluorescence analysis

**Fig. 8:**
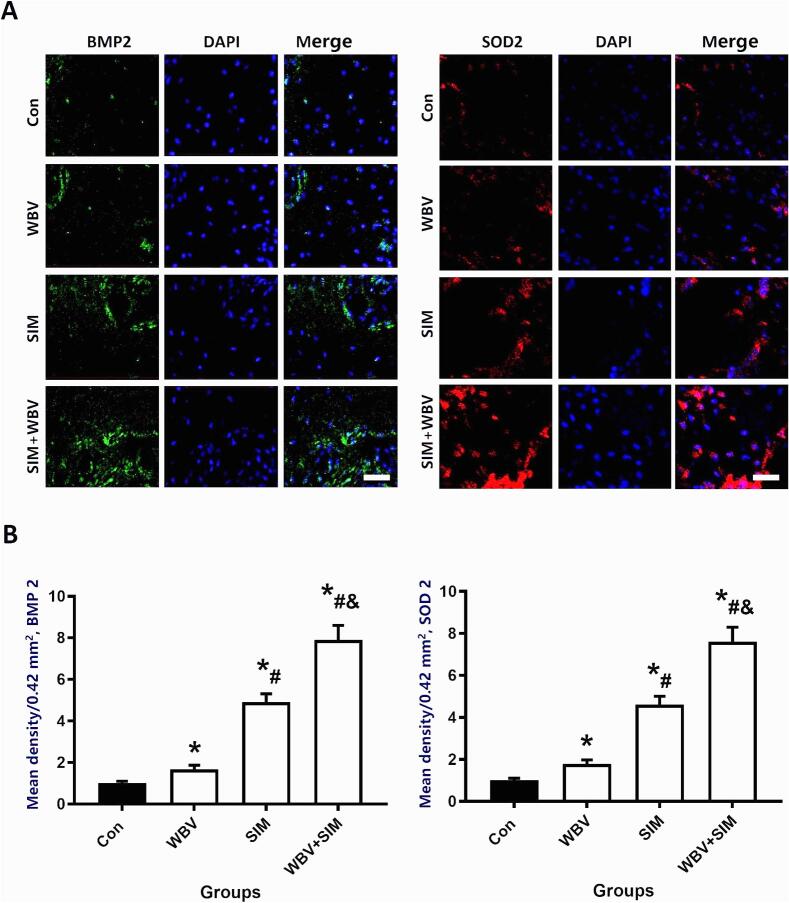
Immunofluorescence detection of BMP2 and SOD2 demonstrates that BMP2 and SOD2 expression in the contralateral femoral epiphysis WBV plus SIM treatment maximally increases BMP2 and SOD2 expression in the contralateral femoral epiphysis. A. Representative images of BMP2 and SOD2 immunofluorescence detection taken at 200 x original magnification in Con group, WBV group, SIM group, and WBV + SIM group. Bar = 5 μm. B. Quantitative results of BMP2 and SOD2 immunofluorescence mean intensity in 0.42 mm^2^ of epiphyseal bone tissue measured per section per animal for each group. *N* = 5.

The results of immunofluorescence labeling of BMP2 and SOD2 in bone tissue were shown in Fig. 8. WBV and SIM interventions significantly concurrently increasing the levels of BMP2 and SOD2(*P* < 0.05) when compared to the results Con group. While both WBV and SIM each significantly increased expression of BMP2 and SOD2, combined therapy of WBV with SIM stimulated the greatest BMP2 and SOD2 protein expression in the contralateral femoral proximal epiphysis.

### 3.6 RT-PCR

**Fig. 9:**
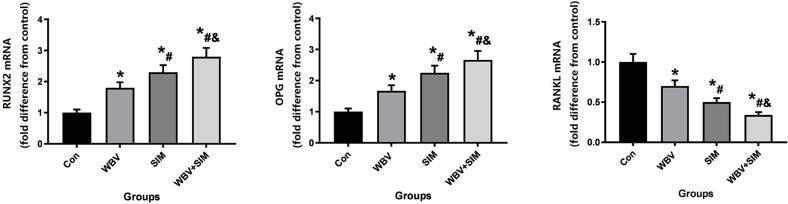
The mRNA levels of Runx2, RANKL, and OPG in the VIO region were obtained from different groups. The values for each were normalized to GAPDH expression. Subsequently, the control values were standardized to be equal to 1, and all other groups were compared in terms of fold difference from the control.

Fig. 9 depicts the assessment of mRNA levels for Runx2, RANKL, and OPG in the vicinity of the implants. In comparison to the Control group (*P* < 0.05), both Runx2 and OPG exhibited significant upregulation in the WBV, SIM, and WBV + SIM groups; whereas RANKL levels were significantly lower in these groups (P < 0.05). Notably, regardless of treatment groups (P < 0.05), the combination therapy of WBV + SIM demonstrated the most pronounced impact on the levels of Runx2, RANKL, and OPG.

## 4 Discussion

This study aimed to investigate the impact of a combined therapy involving whole-body vibration (WBV) and simvastatin (SIM) on osseointegration in elderly individuals, as well as the underlying signaling pathways involved. The administration of SIM and WBV systemically significantly enhanced implant fixation even in cases of poor bone quality. Notably, the combination treatment demonstrated superior pull-out strength for osteoporotic conditions compared to animals treated with either WBV or SIM alone. Furthermore, both WBV and SIM treatments, whether administered individually or in combination, effectively improved bone-to-implant contact and promoted higher relative bone mass around the implants. These interventions also led to increased trabecular thickness and reduced trabecular separation, which are known to enhance biomechanical properties that contribute to preventing bone fragility and reducing fracture risk(Ito et al., 2002). Additionally, our findings revealed that WBV and SIM treatments exhibited inhibitory effects on oxidative stress and inflammation, thereby positively influencing long-term joint prosthesis stability. Further investigation unveiled that both WBV or/and SIM upregulated gene expression of Runx2 and OPG while downregulating RANKL.

In our research, SIM exhibited positive effects on the bone surrounding the implant, leading to a significant increase in parameters such as BV/TV, Tb.Th, Tb.N, and Conn.D when compared to the control group. Furthermore, biomechanical testing confirmed that SIM had a beneficial impact on the stability of the implant. The correlation between micro-CT analysis and biomechanical measurements was strongest for BV/TV and the maximum force required for push-out. Recent investigations have also highlighted promising outcomes of this medication in relation to osteoporosis treatment due to its ability to effectively prevent bone loss (Issa et al., 2015; Xu et al., 2014). Simvastatin promotes bone formation by facilitating mesenchymal cell differentiation into osteoblasts while upregulating bone morphogenetic protein-2 (BMP-2) expression and downregulating osteoblast apoptosis. Additionally, statins may inhibit osteoclast differentiation and activity thereby reducing bone resorption(Oryan et al., 2014). Previous animal studies have evaluated systemic administration of SIM in promoting osseointegration around implants in cases of bone loss(Tao et al., 2016b; Tao et al., 2015). Our findings demonstrate that administering SIM at doses of 25 mg/kg/day enhances osseointegration around implants over a period of 12 weeks by improving trabecular microstructure and enhancing implant fixation.

In the field of orthopedics, it is widely acknowledged that bone adapts its mass and microstructure in response to mechanical loading(Zhao et al., 2019). Whole-body vibration (WBV) loading has been clinically utilized as a non-pharmacological approach for treating osteoporosis due to its ability to accelerate bone formation and fracture healing(Iwamoto et al., 2005; Ma et al., 2016). Previous studies have demonstrated enhanced osseointegration of titanium implants in models of osteoporosis(Akca et al., 2007; Shibamoto et al., 2018). Notably, the specific parameters of WBV, including duration, session distribution, frequency, and amplitude of loading, play crucial roles in influencing peri-implant bone responses(Ogawa et al., 2014; Toru et al., 2011). The results from our study clearly indicate that WBV exhibits significant bone-stimulating effects compared to the OVX group when considering histological analysis, Micro-CT imaging, and push-out tests. This confirms that WBV treatment possesses strong osteogenic potential for promoting implant osseointegration. Consistent with previous research conducted on rat models with ovariectomy-induced osteoporosis(Yi et al., 2015), we also observed a positive impact of WBV on peri-implant bone formation compared to SIM treatment. These findings suggest that WBV represents an effective protocol for enhancing periprosthetic osseointegration among elderly individuals.

Combining whole-body vibration (WBV) therapy with SIM treatment demonstrated enhanced bone formation on implant surfaces in an aged rat model. What could explain the stronger effect of this combination therapy on preserving bone mass and promoting bone formation? One plausible explanation may lie in the distinct mechanisms of action for WBV and SIM. Joint prosthesis loosening is often attributed to oxidative stress and inflammation, which can lead to degradation of the prosthetic device, surrounding bone, and soft tissue, resulting in instability and pain (Esvaran and Conway, 2018; Tsai et al., 2009), Furthermore, oxidative stress and inflammation can exacerbate each other (Yi-Bin et al., 2022), creating a detrimental cycle that accelerates prosthetic device loosening. In our study, we also investigated serum markers of oxidative stress and inflammatory factors. Consistent with our expectations, rats treated with either WBV or SIM exhibited lower levels of oxidative stress and inflammation. Previous research has shown that SIM can reduce bone resorption by inhibiting osteoclast activity while stimulating osteoblast activity to improve bone formation(Tao et al., 2016b; Tao et al., 2015). The upregulation of gene expression for Runx2 and OPG alongside downregulation of RANKL provides a reasonable explanation for this phenomenon. Although specific reasons are not yet known, it is likely that both WBV and SIM contribute to increased local bone formation as well as implant stability through their overall functions. Taking all these findings into account suggests that both SIM and WBV have the potential to decrease levels of oxidative stress and inflammation in aged rats-factors known to promote bone formation while inhibiting bone resorption. Moreover, combining SIM with WBV appears to have a greater impact on these factors ultimately leading to improved osseointegration.

In conclusion, by employing a titanium rod implantation model in aged animals and subjecting them to a 12-week intervention treatment, we have conducted a comprehensive assessment utilizing imaging, histological analysis, and other tests. Our findings unequivocally demonstrate that both Whole Body Vibration (WBV) and Synchronous Implant Motion (SIM) treatments singularly exhibit the capacity to enhance bone mass and promote osseointegration as well as implant stability in aged rats. However, it is worth noting that the synergistic effects of combining the two modalities yield even more favorable outcomes. Our findings suggest that combining physiotherapy (WBV) with drug therapy (SIM) proves beneficial for enhancing implant fixation in aged rats. However, due to the short study time, the limitation of the age of the animals used, and the unknown specific mechanism of the better effect of the combination treatment, we need to further study.

## Funding

This study was supported by a grant from 10.13039/100005622Health Research Project of Anhui Province (AHWJ2023A20554).

## CRediT authorship contribution statement

**Zheng-Bo Qiao:** Writing – review & editing, Writing – original draft, Visualization, Validation, Funding acquisition. **Ming-Zhong Gu:** Software, Project administration, Methodology. **Yu-Wu Wang:** Software, Resources, Methodology, Investigation. **Bin-Bin Ma:** Supervision, Software, Project administration, Investigation. **Shan-Shan Pang:** Funding acquisition, Formal analysis, Data curation, Conceptualization.

## Declaration of competing interest

The authors report no relationships that could be construed as a conflict of interest.

## Data Availability

Data will be made available on request.
